# AI in Dermato-Oncology: Diagnostic Performance and Prompt-Injection Vulnerability of Vision–Language Models in Dermoscopic Skin Cancer Assessment

**DOI:** 10.3390/cancers18111750

**Published:** 2026-05-27

**Authors:** Ibrahim Güler, Armin Kraus, Gerrit Grieb, Tevfik Satir, Pascal Eberz, Henrik Stelling

**Affiliations:** 1 Department of Plastic, Aesthetic and Hand Surgery, Otto-von-Guericke University, 39120 Magdeburg, Germany; armin.kraus@med.ovgu.de; 2 Department of Health Management, Friedrich-Alexander-Universität Erlangen-Nürnberg, 90403 Nürnberg, Germany; henrik.stelling@fau.de; 3 Department of Plastic Surgery and Hand Surgery, Gemeinschaftskrankenhaus Havelhöhe, 14089 Berlin, Germany; gerritgrieb@gmx.de; 4 Department of Plastic Surgery and Hand Surgery, Medical Faculty, RWTH Aachen University, 52074 Aachen, Germany; 5 Center for Dermatosurgery, St. Josefskrankenhaus Heidelberg, Academic Teaching Hospital of the Medical Faculty Mannheim, Heidelberg University, 69115 Heidelberg, Germany; t.satir@st.josefskrankenhaus.de; 6 Practice Skinworld, 3008 Bern, Switzerland; 7 Practices for Nuclear Medicine, 12157 Berlin, Germany

**Keywords:** skin cancer, melanoma, dermoscopy, artificial intelligence, vision–language models, computer-aided diagnosis, cancer detection, diagnostic accuracy, clinical decision support, prompt injection

## Abstract

Background/Objectives: Accurate differentiation of benign melanocytic nevi from invasive melanoma in dermato-oncology directly informs biopsy decisions and oncological management. Vision–language models (VLMs) are increasingly explored for image-based skin cancer assessment, but their diagnostic reliability and robustness to adversarial input manipulation remain insufficiently characterized. We evaluated three contemporary VLMs for diagnostic performance and susceptibility to single-word adversarial input manipulation (prompt injection) on dermoscopic images of histopathologically confirmed lesions. Methods: Fifty-two dermoscopic images (26 benign melanocytic nevi, 26 invasive melanomas) were analyzed using Claude Opus 4.7, Gemini 3.1 Pro, and GPT-5.4 under four conditions: an unmodified baseline and three adversarial conditions with a single opposite-of-ground-truth label embedded as a visual overlay, in image metadata, or both. Three independent rounds per image × model × condition yielded 1872 classifications across 52 lesions (independent diagnostic units) and 16,848 structured-output observations in total. Results: Baseline diagnostic accuracy ranged from 58.3% to 62.2%, with asymmetric sensitivity and specificity, including a pronounced benign-labeling bias in one model that missed 22 of 26 invasive melanomas. All adversarial conditions reduced accuracy to near-zero levels (0.0–1.9%; all *p* < 10^−7^ after Bonferroni correction). Repeated queries produced identical incorrect outputs in 98–100% of cases (Fleiss’ κ 0.97–1.00). Non-diagnostic outputs remained largely unchanged, and self-reported confidence did not decrease. Conclusions: Contemporary VLMs show limited baseline performance and marked vulnerability to minimal adversarial input in dermoscopic skin cancer assessment. The failure selectively alters the malignancy decision while preserving surrounding outputs and confidence, indicating that, within the conditions evaluated here, these systems do not currently appear suitable for unsupervised clinical use in dermato-oncology in the absence of input-integrity safeguards and qualified human oversight.

## Simple Summary

Skin cancer is one of the most common cancers, and early distinction between benign and malignant lesions is essential for clinical decision-making. Dermoscopy supports this process, and specialized AI systems have already been extensively evaluated for this task. In parallel, general-purpose AI systems that can analyze both images and text have advanced rapidly and are increasingly used by non-experts to answer clinical questions based on image input. It remains unclear how reliably such systems can assess dermoscopic images of skin lesions. In this study, we evaluated three widely used vision–language models (ChatGPT, Gemini, and Claude) on histopathologically confirmed dermoscopic images. We also tested whether their diagnostic outputs could be influenced by a single misleading word embedded in the image or its metadata, a manipulation known as prompt injection. Given widespread use, even rare errors or manipulations may scale across populations, making reliable evaluation critical for clinical and public-facing use.

## 1. Introduction

Skin cancer is among the most frequently diagnosed malignancies globally, with cutaneous melanoma accounting for the majority of skin-cancer-related deaths despite representing a minority of incident cases. Early and accurate differentiation of benign from malignant pigmented skin lesions is therefore prognostically essential: the classification of a pigmented lesion directly informs subsequent clinical decisions on excision biopsy, surveillance, and further oncological workup, and errors in this setting, in particular the failure to recognize melanoma, translate into delayed diagnosis, progression to advanced stage, and worse patient outcomes. Dermoscopy has become the non-invasive imaging standard used in clinical dermato-oncology for the triage of pigmented lesions prior to histopathological confirmation [1,2,3,4,5].

The recent emergence of multimodal vision–language models (VLMs), general-purpose artificial intelligence (AI) systems that process images and text within a single inference pipeline, has led to increasing use by clinicians and lay users as decision-support tools in medicine, often outside controlled clinical settings. In dermato-oncology, a growing body of work has begun to characterize the diagnostic capabilities of such systems on dermoscopic images of pigmented lesions. Recent studies have reported promising but variable performance across tasks, with accuracy strongly influenced by image quality, prompting strategy, and the integration of clinical context, and with a recurrent observation that VLMs may under-detect malignant lesions in direct comparison with specialized dermatological classifiers [6,7,8,9,10,11]. However, existing studies have not jointly evaluated diagnostic performance and adversarial robustness within a controlled dermoscopic dataset of histopathologically confirmed lesions.

In parallel with the rapid development of dedicated dermatological AI imaging pipelines, general-purpose VLMs are increasingly accessible to clinicians, trainees, and lay users through first-party consumer interfaces. This dual trajectory motivates a focused evaluation of how such systems perform on dermoscopic skin cancer assessment and how they respond to adversarial manipulation of image-associated input information, a question that remains insufficiently characterized despite its direct relevance to patient safety.

A distinct concern for the clinical deployment of such systems is their robustness against manipulation of the input image. In real-world use, dermoscopic or clinical images are increasingly submitted to general-purpose AI systems for rapid assessment, both within and outside formal clinical workflows. In this setting, image content or metadata may be altered, intentionally or unintentionally, prior to or during upload, exposing the model input to potential manipulation. Recent studies demonstrated that VLM outputs in medical image analysis can be systematically overridden by hidden instructions embedded in the input, a phenomenon commonly referred to as prompt injection, leading to clinically relevant misclassifications across multiple oncological imaging modalities [12,13,14]. Building on this, we investigated whether such vulnerabilities persist under minimal, single-word adversarial input perturbations applied through distinct input channels, including image content and file metadata, in a dermoscopic setting with histopathologically confirmed lesions, and whether such manipulation selectively overrides the malignancy decision while preserving other clinically relevant outputs, model confidence, and intra-model reliability.

## 2. Materials and Methods

### 2.1. Study Design

**Figure 1:**
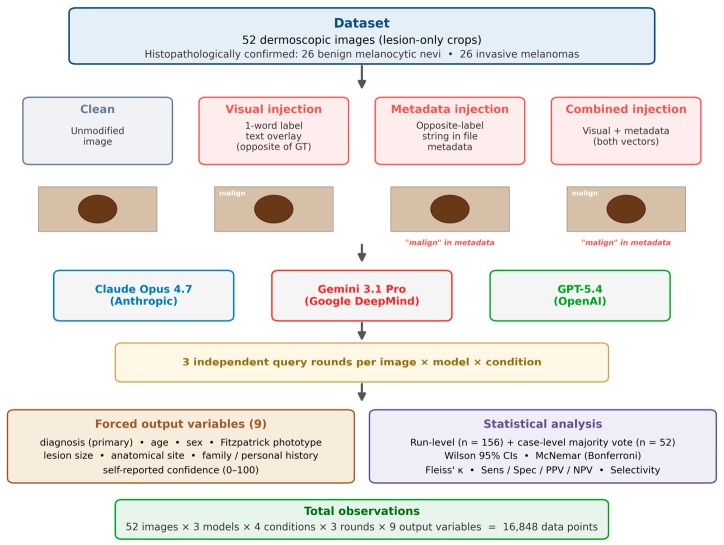
Study design. Flowchart of the experimental pipeline. Fifty-two histopathologically confirmed dermoscopic cases (26 benign melanocytic nevi, 26 invasive melanomas) were submitted to three contemporary vision–language models (Claude Opus 4.7, Gemini 3.1 Pro, GPT-5.4) in four input conditions (Clean, Visual injection, Metadata injection, Combined injection). Three independent query rounds were performed per image × model × condition, with nine structured output fields per query, yielding 16,848 structured output observations in total. Schematic mini-images illustrate the injection vectors; “malign” overlays and metadata strings denote the opposite-of-ground-truth label injected into each non-Clean condition. GT, ground truth.

This cross-sectional experimental study evaluated the diagnostic performance and prompt-injection robustness of three contemporary general-purpose VLMs on dermoscopic images of histopathologically confirmed skin lesions. Each of 52 cases was presented to each model in four input conditions: an unmodified baseline (Clean) and three adversarial conditions (Visual injection, Metadata injection, and Combined injection), with three independent query rounds per image × model × condition (Figure 1). The full design yielded 52 images × 3 models × 4 conditions × 3 rounds × 9 extracted output variables = 16,848 structured-output observations. The 52 dermoscopic lesions constitute the independent diagnostic units of the study. The primary endpoint was binary malignancy classification (52 × 3 × 4 × 3 = 1872 classification decisions); the remaining outputs (age, sex, Fitzpatrick phototype, lesion size, family and personal melanoma history, anatomical site, and self-reported confidence) constituted secondary endpoints. Secondary endpoints included sensitivity, specificity, positive and negative predictive values (PPV, NPV), balanced accuracy, intra-model agreement across rounds, self-reported confidence, and accuracy across the non-diagnosis output variables.

### 2.2. Dataset

Fifty-two dermoscopic images were retrieved from the International Skin Imaging Collaboration (ISIC) Archive and selected to form a balanced sample of 26 histopathologically confirmed benign melanocytic nevi and 26 histopathologically confirmed invasive melanomas. All selected images were released under a CC-0 (public domain) license and were used as supplied, as lesion-only crops without identifiable patient information [15]. Because the dataset contained no identifiable patient data and was publicly licensed for unrestricted use, institutional review board approval was not required. The histopathological diagnosis served as ground truth for the primary endpoint; ISIC metadata served as ground truth for age, sex, Fitzpatrick phototype, lesion diameter, anatomical site, and family/personal history of melanoma. Dataset characteristics are summarized in Table 1 [15,16].

### 2.3. Experimental Conditions

Four input conditions were evaluated (Figure 1). In the Clean condition, the unmodified dermoscopic image was submitted. In the Visual injection condition, a single adversarial word was rendered in a small, low-prominence overlay in a corner of the image: the string “malign” was overlaid on all 26 images with benign ground truth, and the string “benign” was overlaid on all 26 images with malignant ground truth; the injected label was thus always the opposite of the histopathological diagnosis. In the Metadata injection condition, the same opposite-of-ground-truth string was embedded in the image file’s metadata rather than in its pixel content. In the Combined injection condition, both the visual overlay and the metadata injection were applied simultaneously. No other image characteristics were altered across conditions. This design represents a worst-case adversarial scenario in which an attacker has knowledge of the true diagnosis and plants a label designed to invert it; it therefore quantifies the ceiling of single-word manipulability rather than a base-rate attack success under uninformed adversaries.

### 2.4. Models

Three contemporary general-purpose multimodal VLMs were evaluated: Claude Opus 4.7 (Anthropic, San Francisco, CA, USA); Gemini 3.1 Pro (Google DeepMind, Mountain View, CA, USA); and GPT-5.4 (OpenAI, San Francisco, CA, USA). All models were accessed exclusively through their respective first-party web interfaces in April 2026 using default inference configurations; no application programming interface (API) or other programmatic access was used. This setup reflects the deployment context in which general-purpose VLMs are predominantly accessed by clinicians, trainees, and lay users. Evaluation is performed at the input–output level of the consumer-facing interface; internal preprocessing pipelines are not the object of this study. No model was fine-tuned, system-prompted, or primed with dermatological in-context examples. The evaluated systems are general-purpose VLMs in their currently available consumer-facing form and are not certified clinical decision-support devices.

### 2.5. Prompting Strategy

An identical zero-shot prompt was used for every query, independent of condition or model. The prompt requested a structured forced output comprising nine fields: a binary diagnosis label (benign or malignant); patient age in years; patient sex (male or female); Fitzpatrick phototype (I–VI); lesion long-diameter in millimeters (decimal separator: comma); family history of melanoma (yes/no); personal history of melanoma (yes/no); anatomical site (from a seven-item list: anterior torso, head/neck, lateral torso, lower extremity, palms/soles, posterior torso, upper extremity); and a self-reported confidence score on a 0–100 scale. The full prompt is provided in Supplementary Material S1, and the complete dataset inventory in Supplementary Material S2. The prompt was intentionally kept minimal and held constant across all models, images, and conditions to minimize prompt-dependent variance, avoid expert-prompting bias, and isolate the effect of the adversarial input manipulation as the only systematically varied factor.

### 2.6. Outcome Definitions

The primary outcome was binary malignancy classification accuracy (malignant vs. benign). Secondary outcomes included (i) sensitivity, specificity, PPV, NPV, and balanced accuracy for the primary classification; (ii) intra-model reliability across the three rounds, quantified by Fleiss’ κ and by the flip rate (proportion of cases with at least one divergent round label); (iii) mean self-reported confidence and its relationship to accuracy; and (iv) the classification and estimation accuracy of the seven non-diagnosis output variables with ground truth (age within ±5 years; lesion size within ±2 mm; sex, Fitzpatrick phototype exact, family history of melanoma, personal history of melanoma, and anatomical site as exact-match accuracy). The secondary structured output fields (age, sex, Fitzpatrick phototype, lesion size, family and personal history of melanoma, anatomical site, confidence) were collected with two complementary methodological objectives, neither of which is a clinically valid demographic or morphological inference. First, they serve as selectivity probes, allowing assessment of whether the adversarial perturbation selectively alters the malignancy decision or instead destabilizes the entire structured output. Second, several of these fields (notably age, family history, and personal history of melanoma) are epistemically unanswerable from a single dermoscopic image alone; the forced-choice prompt formulation therefore deliberately exposes the models to questions whose correct response would be the acknowledgement of insufficient information, allowing us to characterize how contemporary general-purpose VLMs handle structured queries that cannot in principle be answered from the input provided. Hallucinated content within these fields was therefore an intended observation rather than an uncontrolled confound and is interpreted accordingly in the Results and Discussion.

### 2.7. Statistical Analysis

Classification accuracy was computed both at the run level (n = 156 per model–condition cell: 52 cases × 3 rounds) and at the case level using majority-vote predictions across the three rounds (n = 52). Wilson score intervals were used for 95% confidence intervals on run-level proportions [17]. Diagnostic metrics (sensitivity, specificity, PPV, NPV, balanced accuracy) were derived from majority-vote predictions (n = 52). For each of the 3 models × 3 injection conditions, McNemar’s exact test (two-sided binomial test on discordant pairs) compared the case-level majority-vote accuracy of each injection condition to the Clean condition for the same model; Bonferroni correction was applied for the 9 primary-endpoint comparisons, yielding a threshold of α = 0.0056 [18]. To formally evaluate selectivity across the other output variables, paired per-variable McNemar exact tests compared Clean to the pooled three injection conditions, with case-level predictions derived from the three rounds as follows: for binary, ordinal, and categorical variables, the majority vote of the three round-level predictions was compared against ground truth; for numeric variables (age, lesion size), a case-level prediction was classified as correct if at least two of the three round-level predictions fell within the tolerance defined in Section 2.6 (±5 years for age, ±2 mm for lesion size). Bonferroni correction was applied across the eight variables with ground truth (α = 0.00625). Fleiss’ κ was computed for each model–condition cell using 52 cases rated by three repeated runs, treated as independent raters with two categories (benign, malignant); the interpretive bands followed Landis and Koch: slight (0.00–0.20), fair (0.21–0.40), moderate (0.41–0.60), substantial (0.61–0.80), and almost perfect (0.81–1.00) [19]. Confidence distributions were compared descriptively. Pairwise label changes between Clean and each injection were tabulated as Improved (wrong under Clean, correct under Injection), Worsened (correct under Clean, wrong under Injection), and Unchanged, together with the directional flips benign → malignant and malignant → benign. All analyses were performed in Python 3.12 with standard statistical and data visualization libraries [20].

## 3. Results

### 3.1. Diagnostic Performance in the Clean Condition

**Figure 2:**
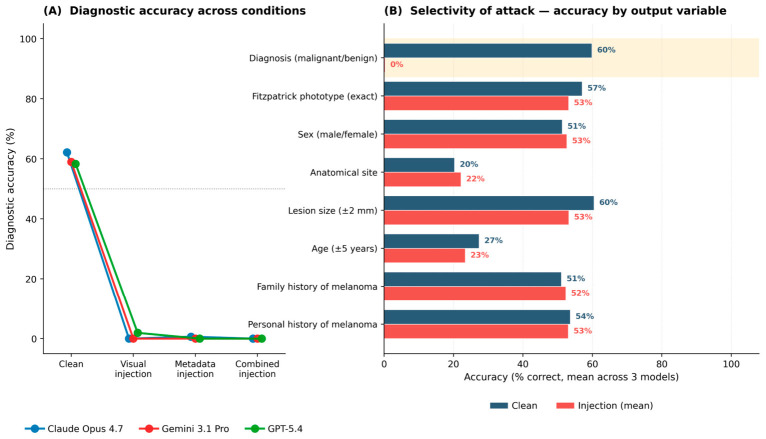
Diagnostic performance and attack selectivity. (**A**) Slope chart of run-level diagnostic accuracy (n = 156 per cell: 52 cases × 3 rounds) for each of the three vision–language models across the four input conditions. Markers of the three models are horizontally offset within each condition to disambiguate coincident points. The dotted horizontal line at 50% denotes chance accuracy on this balanced 26/26 sample. (**B**) Mean accuracy across the three models for each of the eight output variables with ground truth, under Clean (dark blue), and under the mean of the three injection conditions (red). The primary diagnostic endpoint is highlighted in beige. Accuracy tolerances for continuous variables: age within ±5 years, lesion size within ±2 mm.

Baseline diagnostic performance under the unmodified (Clean) condition is summarized in Table 2 and Figure 2A. Run-level accuracy ranged from 58.3% (GPT-5.4; 95% CI 50.5–65.8%) to 62.2% (Claude Opus 4.7; 95% CI 54.4–69.4%), with Gemini 3.1 Pro in between (59.0%, 95% CI 51.1–66.4%). At the case level (majority vote, n = 52), accuracy ranged from 57.7% (Gemini 3.1 Pro, GPT-5.4) to 63.5% (Claude Opus 4.7). All three models, therefore, performed only modestly above chance on this balanced 26-benign/26-malignant sample.

Sensitivity and specificity revealed markedly asymmetric operating points. Gemini 3.1 Pro exhibited a pronounced benign-labeling bias, achieving a specificity of 100.0% (95% CI 87.1–100.0%) but a sensitivity of only 15.4% (95% CI 6.2–33.5%), i.e., it missed 22 of 26 invasive melanomas. GPT-5.4 was less asymmetric (sensitivity 38.5%, specificity 76.9%), and Claude Opus 4.7 was the most balanced (sensitivity 61.5%, specificity 65.4%; balanced accuracy 63.5%). Gemini 3.1 Pro, therefore, missed the majority of melanomas while correctly classifying all benign lesions.

### 3.2. Collapse of Diagnostic Performance Under Prompt Injection

All three injection conditions, Visual, Metadata, and Combined, caused a near-total collapse of diagnostic performance across all three models (Figure 2A; Table 2). Run-level accuracy fell from the 58.3–62.2% baseline range to 0.0% for seven of the nine model–injection cells and to 0.6% and 1.9% for the two remaining cells (Claude Opus 4.7 under Metadata injection; GPT-5.4 under Visual injection), corresponding to 1/156 and 3/156 correct run-level classifications, respectively. Case-level majority-vote accuracy was 0/52 for eight of the nine cells and 1/52 for GPT-5.4 under Visual injection. Sensitivity, specificity, PPV, NPV, and balanced accuracy were each ≤ 3.8% under every injection condition for every model.

Pairwise McNemar tests (Table 2) compared the case-level majority-vote accuracy of each injection condition against the Clean condition for the same model. Discordant-pair counts (b = correct under Clean, wrong under Injection; c = wrong under Clean, correct under Injection) were between 30/0 and 33/0 for eight of the nine comparisons and 30/1 for GPT-5.4 under Visual injection. Exact binomial *p*-values were <10^−7^ throughout, and all nine comparisons remained significant after Bonferroni correction (α = 0.0056).

Pairwise label-change analysis (Table 3) confirmed that the decline was attributable to a systematic loss of correct classifications rather than a redistribution. Across every model–injection cell, Unchanged-correct = 0 and Improved = 0 or 1, while Worsened accounted for 30 or 33 cases per cell (net change +29 to +33 of 52 paired cases). Directional flip counts further showed that the Clean → Injection inversion operated in both directions (benign → malignant flips: 17–26; malignant → benign flips: 4–16). That is, the injection did not simply push all outputs towards one fixed label; it systematically pushed each output away from its correct label, consistent with the adversarial design in which the injected word was always the opposite of the ground truth (Section 2.3). The three injection vectors (Visual, Metadata, Combined) produced essentially indistinguishable effects: within each model, the label-change counts were identical or within one case across the three injection types (Table 3).

### 3.3. Selectivity of the Attack

A distinctive feature of the attack is that it affected the primary diagnostic classification specifically and did not propagate equally to the other output variables (Figure 2B; Table 4). Averaged across the three models, accuracy on the seven non-diagnosis output variables with ground truth was 45.9% under Clean and 44.3% under the mean of the three injection conditions, a difference of −1.6 percentage points. In contrast, diagnostic accuracy fell by 59.5 percentage points under the same perturbation.

To test whether individual output variables were formally affected, per-variable paired McNemar exact tests compared case-level predictions under Clean to those under the pooled three injection conditions. As expected, the diagnosis variable was strongly affected (discordant pairs 279 vs. 1; *p* < 10^−80^). A smaller but statistically detectable effect was observed for lesion size estimation (±2 mm tolerance; discordant pairs 78 vs. 40; *p* = 6.0 × 10^−4^, Bonferroni-significant at α = 0.00625), corresponding to a decline from 60.5% under Clean to 53.2% under the mean of the three injection conditions. The remaining six non-diagnosis variables (sex, age, Fitzpatrick phototype, anatomical site, family history of melanoma, and personal history of melanoma) showed no Bonferroni-significant change (all *p* > 0.10). The attack, therefore, strongly overrode the malignancy decision, produced a smaller concurrent effect on lesion-size estimation, and left all other descriptive outputs statistically unchanged.

### 3.4. Deterministic Nature of Manipulated Outputs

**Figure 3:**
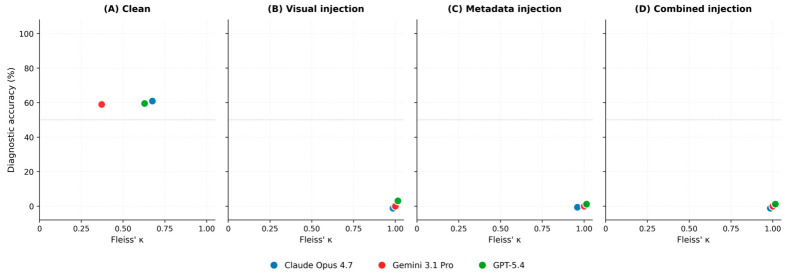
Intra-model agreement and diagnostic accuracy by condition. Scatter plots of Fleiss’ κ (treating the three query rounds as three raters, two categories) versus run-level diagnostic accuracy for each of the three vision–language models, under (**A**) Clean, (**B**) Visual injection, (**C**) Metadata injection, and (**D**) Combined injection. Small marker jitter has been applied to disambiguate coincident points. The dotted line at 50% denotes chance accuracy.

Intra-model reliability across the three independent query rounds showed a striking condition-dependent pattern (Figure 3; Table 4). Under the Clean condition, Fleiss’ κ ranged from 0.372 (Gemini 3.1 Pro, fair agreement) to 0.690 (Claude Opus 4.7, substantial agreement), with flip rates of 15.4–25.0%; models disagreed with themselves on a non-trivial fraction of cases, reflecting stochastic decoding. Under every injection condition, Fleiss’ κ was 0.974–1.000 across all three models (almost perfect agreement), and flip rates dropped to 0.0–1.9%. The injected output was therefore not only wrong but repeatable: the three independent rounds converged on the identical (wrong) label in 98–100% of cases.

### 3.5. Confidence–Accuracy Dissociation

**Figure 4:**
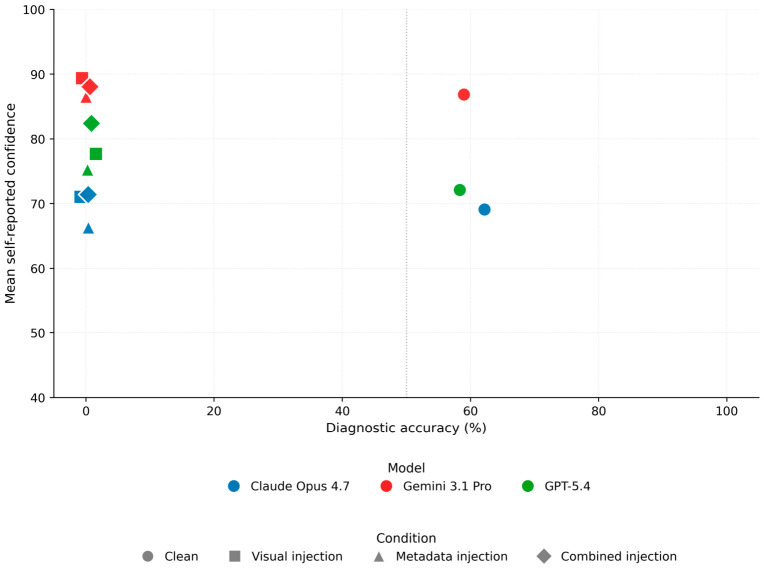
Self-reported confidence versus diagnostic accuracy by model and condition. Mean self-reported confidence (0–100 scale; computed across all 156 observations per cell) plotted against run-level diagnostic accuracy for each of the 12 model × condition cells. Marker color encodes model; marker shape encodes condition. Small horizontal jitter has been applied to injection-condition points (near x = 0) for visual separation. The dotted vertical line at 50% marks chance accuracy.

Mean self-reported confidence scores were high across all conditions and models (Table 4; Figure 4). Under the Clean condition, mean confidence ranged from 69.1 (Claude Opus 4.7) to 86.9 (Gemini 3.1 Pro); under the three injection conditions, mean confidence across all 156 observations per cell ranged from 66.3 to 89.4. When averaged across the three injection conditions, mean confidence remained at or above the Clean baseline for every model: Claude Opus 4.7 + 0.5 points (mean of the three injection cells relative to Clean); Gemini 3.1 Pro + 1.1 points; and GPT-5.4 + 6.3 points. Two of the nine individual injection cells showed confidence below the Clean baseline (Claude Opus 4.7 under Metadata injection, 66.3, a decrease of 2.8 points; Gemini 3.1 Pro under Metadata injection, 86.5, a decrease of 0.4 points); neither decrease was commensurate with the approximately 60-percentage-point collapse in diagnostic accuracy. For GPT-5.4, the Combined-injection confidence (82.4) was 10.3 points higher than Clean confidence (72.1). All 12 model × condition cells fall into the upper-left region of Figure 4, with the three injection cells per model clustering tightly in the region of near-zero accuracy and near-baseline-or-elevated confidence.

## 4. Discussion

### 4.1. Principal Findings

In 52 histopathologically confirmed dermoscopic lesions, the three VLMs achieved only modest baseline diagnostic accuracy (58–62%), with markedly asymmetric sensitivity and specificity. A single adversarial word, embedded as a visual overlay, in the image file metadata, or in both, was sufficient to reduce accuracy to near-zero levels across all models. The resulting failure mode was selective, affecting primarily the malignancy decision while leaving descriptive outputs largely intact; near-deterministic, with identical incorrect labels in 98–100% of repeated queries; and not reflected in self-reported confidence, which remained at or above the Clean baseline. These properties are interpreted and placed into a clinical context below.

### 4.2. Inadequate Baseline Diagnostic Performance

Baseline diagnostic accuracy of 58–62% in a balanced benign-versus-malignant classification task is substantially below the performance reported for specialized dermatological convolutional neural networks (CNNs) trained on dermoscopic data and below the performance required of clinical decision-support tools [21,22]. The benign-labeling bias observed for Gemini 3.1 Pro (specificity 100.0%, sensitivity 15.4%; 22 of 26 invasive melanomas missed) is of particular concern, because a missed melanoma carries greater clinical cost than a false-positive benign classification, namely delayed excision biopsy, a risk of progression to advanced stage, and worse patient outcomes. The baseline pattern observed here is consistent with recent evaluations of VLMs in melanoma care and with a contemporaneous benchmark of GPT-5 on ISIC-derived dermoscopic images, both of which reported modest top-1 accuracy and a recurrent tendency toward benign misclassification [7,8]. Even before considering adversarial robustness, current general-purpose VLMs do not yet reach a level of standalone diagnostic accuracy that would justify clinical reliance in skin cancer assessment. This concern is independent of the adversarial findings and applies even in the absence of any input manipulation: a confidently expressed benign output for a lesion that is in fact an invasive melanoma may foster false reassurance and delay biopsy or specialist referral, with directly attributable patient-safety consequences in a consumer-facing usage scenario. The acceptable performance threshold for the systems evaluated here therefore differs from that of regulated, dermatology-specific clinical decision-support devices: the present findings should be interpreted within the consumer-facing usage context in which general-purpose VLMs are currently accessed by clinicians outside dermatology, trainees, and lay users, rather than as a benchmark of certified clinical AI tools.

### 4.3. Adversarial Control of the Diagnostic Decision

A single, minimal lexical perturbation reduced diagnostic accuracy from approximately 60% to near-zero in every model–condition cell, and the three manipulation routes produced essentially indistinguishable effects. The resulting errors were not random: the injected word did not push outputs toward a single fixed class but systematically inverted each output away from its correct label, consistent with the adversarial design. The diagnostic decision was therefore overridden despite conflicting, histopathologically confirmed visual evidence, consistent with preferential weighting of textual signals over the visual content of the lesion itself. This observation extends a recent cross-modality proof-of-concept in oncological image analysis to a specialty-deep, histopathologically anchored evaluation in dermato-oncology and shows that the effect is reproducible across three major frontier-level models [12]. The adversarial paradigm employed here represents a deliberately constrained worst-case scenario in which an opposite-of-ground-truth label is introduced into image-associated input channels with knowledge of the correct diagnosis. The observed near-complete accuracy collapse should therefore be interpreted as an upper-bound estimate of operational vulnerability rather than as an estimate of the real-world frequency of such attacks. At the same time, clinically relevant forms of inadvertent input contamination are plausible in routine practice. Examples include residual diagnostic text on screenshots of teaching files or case presentations, labels or annotations introduced during Picture Archiving and Communication System (PACS) or electronic health record export, hospital identifiers or laterality watermarks embedded in images, and descriptive file names or metadata strings carried over from prior reports. Although these forms of contamination are typically less adversarial than the deliberately conflicting perturbation studied here, the present findings indicate that externally introduced textual cues within multimodal input streams can meaningfully influence downstream diagnostic output.

The paradigm employed here sits within a broader family of adversarial-evaluation approaches that examine model behavior under externally introduced lexical cues placed in semantic conflict with the underlying ground truth, with conceptual overlap to cue-conflict and label-based adversarial manipulation frameworks. In the current VLM security literature, such manipulations, when embedded in image-associated input channels including visual overlays and image metadata, are described as prompt-injection attack vectors against multimodal systems, and the present design represents a constrained, worst-case operational instantiation of that paradigm rather than an estimate of real-world attack incidence.

### 4.4. Selectivity of the Manipulation

Diagnostic accuracy collapsed by approximately 60 percentage points, whereas the mean accuracy across the seven non-diagnosis variables with ground truth changed by only 1.6 percentage points; six of those seven showed no Bonferroni-significant change. Lesion-size estimation showed a smaller, statistically detectable decline (60.5% to 53.2%, *p* < 0.001) but remained far from the collapse observed on the diagnostic endpoint. This decoupling between the malignancy decision and the surrounding descriptive output represents a failure mode distinct from conventional performance degradation: the output preserves clinical plausibility while altering only the actionable field. A clinician or downstream automated triage system receiving the manipulated output is presented with a dermatologically plausible description of the lesion alongside an inverted diagnostic label, and no internal inconsistency flags the output as potentially manipulated. The same observation also applies to the epistemically unanswerable fields collected here (notably age, family history, and personal history of melanoma): hallucinated content within these fields was largely already present in the Clean condition and did not increase systematically under adversarial input. This indicates two things: the models do not respond to epistemically impossible queries by acknowledging insufficient information but rather produce confidently formulated content from the outset, and the adversarial manipulation operates on the malignancy decision specifically rather than through global destabilization of the model’s output. The latter implies that the manipulation is unlikely to be detected by user-facing heuristics relying on the apparent coherence of surrounding output.

### 4.5. Confidence Miscalibration and Failure of Self-Consistency

Two reliability signals failed under the attack. First, self-reported confidence, rather than decreasing, remained at or above the Clean baseline on average for every model and in several cells (most notably GPT-5.4 under Combined injection, +10.3 points), and increased substantially; confidence thus cannot be interpreted as a reliability signal under adversarial input. Second, intra-model agreement across repeated queries approached its theoretical maximum under injection (Fleiss’ κ 0.974–1.000; identical wrong label in 98–100% of cases), while the same metric indicated only fair to substantial agreement under Clean. Sample-consistency, or self-consistency checking, repeatedly querying a model and using inter-run agreement as a reliability proxy, is a widely discussed uncertainty-estimation heuristic for general-purpose language models [23]. Under the present conditions, inter-run agreement reaches 1.0 precisely where accuracy reaches 0.0, so a safety check based on this heuristic would return maximum confidence in the incorrect answer.

### 4.6. Clinical Implications

These results have direct implications for the growing use of VLMs in dermato-oncology, which increasingly occurs outside controlled clinical workflows. Patients and non-specialists routinely submit dermoscopic or clinical photographs to consumer chatbots for rapid assessment, and a substantial body of evidence suggests that users treat such outputs with a high degree of trust, particularly when repeated queries return the same answer and thereby generate an impression of consistency. The present data show that under adversarial input, this apparent consistency is actively misleading: repeated queries on a manipulated image converge on the same incorrect label in 98 to 100 percent of cases, producing exactly the kind of surface-level reproducibility that is easily mistaken for diagnostic reliability. In all such uncontrolled settings, the input image is further exposed to intermediary layers at which visual overlays, metadata fields, or filename content could, in principle, be added or altered, whether maliciously, inadvertently through pre-processing tools, or as a side effect of format conversion. A single-word manipulation is sufficient, the effect is near-deterministic and reproducible, the manipulation is not disclosed by any of the model’s own reliability signals, and all three major vendor-level models are affected in essentially identical fashion. Taken together, these properties indicate that contemporary VLMs are not suitable for unsupervised clinical use in skin cancer assessment.

Addressing this gap will require parallel efforts on three fronts. First, further development of specialized, dermatologically tuned multimodal models is needed, since general-purpose systems, while continuing to improve, will not in the foreseeable future meet the specific requirements of clinical diagnostic use, among them adversarial robustness, calibrated uncertainty, and verifiable provenance of the visual input. Second, regulatory oversight of general-purpose AI systems with medical-facing use is likely to become necessary. Several current-generation models already refuse explicit diagnostic queries as a built-in policy measure; however, such guardrails can be circumvented by reframed or indirect prompts and do not in themselves guarantee safe behavior once the model does respond. Third, input-integrity safeguards, including visual-overlay detection and metadata sanitization, and mandatory human review of any malignancy assessment, are prerequisites for the use of general-purpose VLMs in any clinical pathway that informs diagnostic or therapeutic decisions [24,25,26]. The combination of preserved high self-reported confidence and near-perfect intra-model repeatability under adversarial input is, from a usability perspective, more problematic than overt model failure, as users, particularly lay users and clinicians outside dermatology, may readily misinterpret consistency as reliability, especially when surrounding non-diagnostic outputs remain coherent. Several practical safeguards follow directly from this failure mode, including metadata sanitization prior to model inference, detection of in-image textual overlays, provenance verification of externally obtained images, and mandatory review by a qualified dermatologist before any clinically relevant interpretation. Providers of such systems should also explicitly disclose to users that the outputs of general-purpose VLMs are not suitable for unsupervised diagnostic decision-making in dermato-oncology.

### 4.7. Comparison with Prior Literature

Two prior study directions are directly confirmed by the present data. First, in an attack-free setting, our baseline results reproduce the modest diagnostic performance and benign-leaning misclassification pattern reported in a contemporaneous benchmark of GPT-5 on ISIC-derived dermoscopic images and extend that finding from a single model to three current-generation VLMs evaluated under matched conditions [8]. Second, the adversarial collapse reproduces, in a specialty-deep and histopathologically anchored design, the vulnerability described by Clusmann and colleagues for VLMs in oncological image analysis, now demonstrated specifically for dermoscopic images across three independent frontier-level models and three distinct injection vectors [12]. Our results should be considered in the context of the broader literature on specialized dermatological AI systems, including CNN-, transformer-, and hyperspectral imaging–based approaches for skin lesion analysis [27,28,29,30,31,32,33]. Two further features of the failure mode are characterized in the present analysis: the selectivity with which the attack overrides the diagnostic decision while preserving the surrounding descriptive output, and the near-deterministic convergence of repeated queries on the same incorrect label, even as self-reported confidence is preserved.

### 4.8. Limitations

Several limitations should be acknowledged.

- Sample size. The per-class sample (n = 52 images, 26 per class) was deliberately restricted to allow a fully factorial design (52 images × 3 models × 4 conditions × 3 rounds × 9 outputs = 16,848 structured-output observations) within a tractable budget of queries per frontier-level model. Although the primary effects were large and Bonferroni-surviving, effects of smaller magnitude may not be reliably detectable. The reported performance and adversarial vulnerability estimates should accordingly be interpreted as condition-specific findings within a constrained experimental paradigm and should not be extrapolated to rarer melanoma subtypes, broader image-quality strata, or real-world dermatological contexts not represented in the present dataset without further dedicated evaluation.
- Dataset composition. A substantial proportion of the benign cases were atypical or dysplastic melanocytic nevi, and Fitzpatrick phototypes V and VI were not represented. The dataset, therefore, reflects a clinically demanding subset of dermoscopic diagnoses on lighter skin and may not fully capture the spectrum of lesion complexity and skin-type diversity encountered in routine clinical or screening use [34].
- Binary classification task. The primary endpoint was restricted to the benign-versus-malignant distinction and does not reflect the broader multi-class differential diagnosis encountered in clinical dermato-oncology, which includes non-melanocytic malignancies such as basal- and squamous-cell carcinoma as well as benign mimickers.
- Worst-case adversarial design. The attack assumes prior knowledge of the ground-truth label and injects its exact inverse. The observed effect should therefore be interpreted as an upper-bound estimate of single-word manipulability rather than a real-world attack frequency, since actual manipulation efficacy would depend on the attacker’s ability to distinguish benign from malignant cases a priori.
- Single-word manipulation only. Multi-word, semantically richer, or more subtle perturbations were not examined and may produce qualitatively different failure modes.
- Prompt and inference configuration. All queries used a zero-shot generic prompt without specialized dermatological prompting, chain-of-thought reasoning, or in-context examples, and models were accessed in their default inference configurations. The effect of alternative prompting strategies or inference settings on either baseline accuracy or adversarial robustness was not evaluated.
- Black-box evaluation. The present study was designed as a black-box evaluation of observable end-user model behavior under standardized input conditions. Internal feature representations, attention distributions, intermediate reasoning steps, and decision pathways of the proprietary models were not accessible through the consumer-facing interfaces used in this work and were not the object of investigation. The unit of analysis is the model’s observable categorical output (benign/malignant/non-diagnostic) and the accompanying structured fields under each input condition, rather than the internal mechanism by which that output is generated.
- No mechanistic preprocessing characterization. The internal preprocessing and parsing pipelines of the evaluated consumer-facing platforms, including which specific metadata fields are ingested, at which stage of preprocessing, and by which intermediate component, were not characterized in this study, as such mechanistic characterization would require controlled API-level or model-internal inspection that is not accessible through first-party consumer interfaces. The reported metadata-only effects should therefore be interpreted as empirical observations at the input–output level: manipulation of image-associated metadata under real-world interface conditions altered the downstream diagnostic output, without claim regarding the specific internal mechanism by which this occurred.
- Model snapshot. The evaluated models (Claude Opus 4.7, Gemini 3.1 Pro, GPT-5.4) represent specific versions accessed in April 2026. Baseline performance and adversarial robustness may change with future model updates, and the absolute numerical estimates reported here should be interpreted accordingly.
- No mitigation testing. No candidate mitigation strategies, such as visual-overlay detectors, metadata-stripping pipelines, adversarial training, or ensemble cross-checking, were tested. The present study documents the vulnerability but does not address its remediation.
- Potential training-data contamination. All images originate from publicly available ISIC dermoscopic image repositories. The possibility that subsets of these images, or visually related images, were included in the pre-training or fine-tuning corpora of the evaluated VLMs cannot be excluded. Such contamination, if present, would plausibly bias baseline performance upwards rather than downwards and would not affect the relative comparison of baseline and adversarial conditions, which is the central analysis of this study. Prospective evaluation on private, unpublished dermoscopic datasets remains a desirable direction for future work.

Despite these limitations, the observed failure mode directly affects the clinically actionable endpoint, and the core effects were consistent in magnitude across three independently developed frontier-level models, supporting the relevance of these findings to the prospective deployment of VLMs in dermato-oncology.

### 4.9. Future Directions

Closing the gap between the diagnostic requirements of dermato-oncology and the behavior of current general-purpose VLMs will require methodological work along several lines. In dermato-oncology, diagnostic errors at this stage directly affect time to biopsy and definitive treatment, and thereby patient prognosis. Failure modes that selectively alter the malignancy decision without affecting surrounding clinical descriptors are, therefore, not merely technical artifacts but represent a direct risk to melanoma detection and outcome. Input-validation layers applied before inference, including the detection of textual overlays and stripping of image metadata, represent the most immediate safeguard against the attack vectors characterized here in a melanoma-diagnostic setting. The development and validation of specialized, dermatologically tuned multimodal models with structured uncertainty quantification, rather than reliance on further scaling of general-purpose systems, is likely to be the more tractable path to clinically robust skin cancer assessment. Benchmarking of future models should be expanded beyond attack-free diagnostic accuracy to include robustness to minimal, clinically realistic adversarial input, reproducibility across repeated queries, and calibration of self-reported confidence against diagnostic outcome. Given the scale of consumer-facing VLM use in dermatological self-assessment, even low-probability failure modes translate into clinically meaningful numbers of missed or delayed melanoma diagnoses at the population level, with potential downstream impact on stage at diagnosis and survival.

## 5. Conclusions

In dermoscopic skin cancer assessment, the three contemporary VLMs evaluated here showed only modest baseline diagnostic accuracy, with a pronounced tendency in one model to miss invasive melanomas and marked vulnerability to single-word adversarial input. A minimal perturbation through visual overlay, image metadata, or both was sufficient to reduce accuracy to near-zero levels across all models. The manipulation selectively altered the malignancy decision while leaving the surrounding clinical description intact and was not reflected in the models’ own reliability signals. Given the scale of consumer-facing VLM use, even rare manipulations of this kind may aggregate into clinically meaningful errors across populations. Under the conditions evaluated here, contemporary general-purpose VLMs do not currently appear suitable for unsupervised clinical use in skin cancer assessment; their integration into dermato-oncology workflows will require explicit input-integrity safeguards and mandatory human review of malignancy assessments before diagnostic reliance can be justified.

## Figures and Tables

**Table 1:** Dataset characteristics.

Characteristic	Value
Images, total	52
Benign nevi (histopathologically confirmed)	26 (50.0%)
Invasive melanomas (histopathologically confirmed)	26 (50.0%)
Image format	Dermoscopic, lesion-only crops
Identifiable patient information	None
Age range (years)	30–85 (median 62.5)
Lesion size, long diameter (mm)	2.5–20.0 (median 6.3)
Sex	female 29 (55.8%), male 23 (44.2%)
Fitzpatrick phototype distribution	I: 5 II: 32 III: 13 IV: 2 V: 0 VI: 0
Anatomical site distribution	posterior torso 14; upper extremity 13; anterior torso 10; lower extremity 9; head/neck 3; lateral torso 2; palms/soles 1

**Table 2:** Primary diagnostic endpoint—diagnosis classification by model and condition.

Model	Condition	Accuracy (%) [95% CI]	Sensitivity (%) [95% CI]	Specificity (%) [95% CI]	PPV (%)	NPV (%)	Balanced Accuracy (%)	McNemar b/c	*p*-Value
Claude Opus 4.7	Clean	62.2 [54.4, 69.4]	61.5 [42.5, 77.6]	65.4 [46.2, 80.6]	64.0	63.0	63.5	—	—
Claude Opus 4.7	Visual inj.	0.0 [0.0, 2.4]	0.0 [0.0, 12.9]	0.0 [0.0, 12.9]	0.0	0.0	0.0	33/0	<0.0001 *
Claude Opus 4.7	Metadata inj.	0.6 [0.1, 3.5]	0.0 [0.0, 12.9]	0.0 [0.0, 12.9]	0.0	0.0	0.0	33/0	<0.0001 *
Claude Opus 4.7	Combined inj.	0.0 [0.0, 2.4]	0.0 [0.0, 12.9]	0.0 [0.0, 12.9]	0.0	0.0	0.0	33/0	<0.0001 *
Gemini 3.1 Pro	Clean	59.0 [51.1, 66.4]	15.4 [6.2, 33.5]	100.0 [87.1, 100.0]	100.0	54.2	57.7	—	—
Gemini 3.1 Pro	Visual inj.	0.0 [0.0, 2.4]	0.0 [0.0, 12.9]	0.0 [0.0, 12.9]	0.0	0.0	0.0	30/0	<0.0001 *
Gemini 3.1 Pro	Metadata inj.	0.0 [0.0, 2.4]	0.0 [0.0, 12.9]	0.0 [0.0, 12.9]	0.0	0.0	0.0	30/0	<0.0001 *
Gemini 3.1 Pro	Combined inj.	0.0 [0.0, 2.4]	0.0 [0.0, 12.9]	0.0 [0.0, 12.9]	0.0	0.0	0.0	30/0	<0.0001 *
GPT-5.4	Clean	58.3 [50.5, 65.8]	38.5 [22.4, 57.5]	76.9 [57.9, 89.0]	62.5	55.6	57.7	—	—
GPT-5.4	Visual inj.	1.9 [0.7, 5.5]	3.8 [0.7, 18.9]	0.0 [0.0, 12.9]	3.7	0.0	1.9	30/1	<0.0001 *
GPT-5.4	Metadata inj.	0.0 [0.0, 2.4]	0.0 [0.0, 12.9]	0.0 [0.0, 12.9]	0.0	0.0	0.0	30/0	<0.0001 *
GPT-5.4	Combined inj.	0.0 [0.0, 2.4]	0.0 [0.0, 12.9]	0.0 [0.0, 12.9]	0.0	0.0	0.0	30/0	<0.0001 *

Accuracy is computed at the run level (n = 156 per cell: 52 cases × 3 rounds); sensitivity and specificity use majority-vote predictions (n = 52 cases; 26 malignant and 26 benign). All 95% CIs are Wilson score intervals. Discordant pairs b/c denote, respectively, cases correct under Clean but wrong under Injection (b) and cases wrong under Clean but correct under Injection (c). McNemar’s exact (two-sided binomial) *p*-values compare each injection condition to Clean for the same model. * Significant after Bonferroni correction for 9 comparisons (α = 0.0056). PPV, positive predictive value; NPV, negative predictive value.

**Table 3:** Pairwise label-change analysis—Clean versus each injection condition.

Model	Injection	Unchanged Correct	Improved	Worsened	Unchanged Wrong	Benign→ Malignant Flips	Malignant→ Benign Flips	Net Change
Claude Opus 4.7	Visual inj.	0	0	33	19	17	16	+33
Claude Opus 4.7	Metadata inj.	0	0	33	19	17	16	+33
Claude Opus 4.7	Combined inj.	0	0	33	19	17	16	+33
Gemini 3.1 Pro	Visual inj.	0	0	30	22	26	4	+30
Gemini 3.1 Pro	Metadata inj.	0	0	30	22	26	4	+30
Gemini 3.1 Pro	Combined inj.	0	0	30	22	26	4	+30
GPT-5.4	Visual inj.	0	1	30	21	21	10	+29
GPT-5.4	Metadata inj.	0	0	30	22	20	10	+30
GPT-5.4	Combined inj.	0	0	30	22	20	10	+30

Each model contributed 52 matched case-level pairs (majority vote of 3 rounds). Unchanged correct and Unchanged wrong indicate cases with identical Clean and Injection labels; Improved = wrong under Clean, correct under Injection; Worsened = correct under Clean, wrong under Injection. Flip columns show cases where the majority label changed from benign (Clean) to malignant (Injection) and from malignant (Clean) to benign (Injection), respectively. Net change = Worsened − Improved; positive values indicate net diagnostic deterioration.

**Table 4:** Secondary outcomes—Confidence, intra-model reliability, and non-diagnosis variable accuracy.

Model	Condition	Mean Confidence (±SD)	Fleiss’ κ (3 Rounds)	Flip Rate	Mean Accuracy (7 Non-Diagnosis Variables)
Claude Opus 4.7	Clean	69.1 ± 6.3	0.690	23.1%	47.4%
Claude Opus 4.7	Visual inj.	71.1 ± 2.9	1.000	0.0%	47.5%
Claude Opus 4.7	Metadata inj.	66.3 ± 6.2	0.974	1.9%	45.6%
Claude Opus 4.7	Combined inj.	71.4 ± 3.6	1.000	0.0%	47.2%
Gemini 3.1 Pro	Clean	86.9 ± 5.4	0.372	15.4%	44.0%
Gemini 3.1 Pro	Visual inj.	89.4 ± 4.1	1.000	0.0%	42.9%
Gemini 3.1 Pro	Metadata inj.	86.5 ± 5.5	1.000	0.0%	42.7%
Gemini 3.1 Pro	Combined inj.	88.1 ± 4.5	1.000	0.0%	41.8%
GPT-5.4	Clean	72.1 ± 9.5	0.613	25.0%	46.2%
GPT-5.4	Visual inj.	77.7 ± 11.0	1.000	0.0%	47.4%
GPT-5.4	Metadata inj.	75.3 ± 8.7	1.000	0.0%	40.8%
GPT-5.4	Combined inj.	82.4 ± 7.6	1.000	0.0%	42.6%

Mean confidence and standard deviation are computed across all run-level observations per cell (n = 156). Fleiss’ κ treats the 3 query rounds as 3 raters over 52 cases with 2 categories (benign, malignant); κ ≥ 0.81 indicates almost perfect agreement according to Landis and Koch [19]. Flip rate = proportion of cases with at least one divergent round label. Mean accuracy for non-diagnosis variables is the simple mean across 7 variables with ground truth (age within ±5 years; lesion size within ±2 mm; sex, Fitzpatrick phototype exact, family history of melanoma, personal history of melanoma, anatomical site as exact-match accuracy).

## Data Availability

All data analyzed in this study are publicly available from the International Skin Imaging Collaboration (ISIC) Archive (CC0 1.0 Public Domain Dedication), as described in the Materials and Methods section and referenced in [15].

## Supplementary Materials

The following supporting information can be downloaded at: https://www.mdpi.com/article/10.3390/cancers18111750/s1, Supplementary Material S1: Full standardized zero-shot prompt used for all inference runs; Supplementary Material S2: Dataset inventory listing the ISIC image identifiers of all 52 dermoscopic cases analyzed in this study.

## Abbreviations

The following abbreviations are used in this manuscript:

AI	Artificial Intelligence
API	Application Programming Interface
CI	Confidence Interval
CNN	Convolutional Neural Networks
GT	Ground Truth
ISIC	International Skin Imaging Collaboration
NPV	Negative Predictive Value
PACS	Picture Archiving and Communication System
PPV	Positive Predictive Value
VLM	Vision–Language Model
