# Supplementary material for: AI in Dermato-Oncology: Diagnostic Performance and Prompt-Injection Vulnerability of Vision–Language Models in Dermoscopic Skin Cancer Assessment
Source: Cancers (Basel). 2026 May 27;18(11):1750. doi: 10.3390/cancers18111750 (PMC13255884; doi:10.3390/cancers18111750)
Supplement: Supplementary file 1 [file cancers-18-01750-s001.zip › S2 Dataset inventory.pdf]

Supplementary Material S2: Dataset inventory listing the ISIC image identifiers of all 52 dermoscopic cases analyzed in this study.

ISIC_0211399
ISIC_0437256
ISIC_0503442
ISIC_1279356
ISIC_2382511
ISIC_2712615
ISIC_3332716
ISIC_3467133
ISIC_3580083
ISIC_3596982
ISIC_3640764
ISIC_3868516
ISIC_3987802
ISIC_4032749
ISIC_4733754
ISIC_4850167
ISIC_5133582
ISIC_5241686
ISIC_5336115
ISIC_5531260
ISIC_5549257
ISIC_5991710
ISIC_6767333
ISIC_7376567
ISIC_7581139
ISIC_7859727
ISIC_0202119
ISIC_0235696
ISIC_0261253
ISIC_0648545
ISIC_2663597
ISIC_2757332
ISIC_2867416
ISIC_4097868
ISIC_4223497
ISIC_5923557
ISIC_6410859
ISIC_6556373
ISIC_6668093
ISIC_6714923

ISIC_6792899
ISIC_6892286
ISIC_7412327
ISIC_7747712
ISIC_8256480
ISIC_8308321
ISIC_8671119
ISIC_8856365
ISIC_8907509
ISIC_9159477
ISIC_9214649
ISIC_9881460
